# Rational development and application of biomarkers in the field of autoimmunity: A conceptual framework guiding clinicians and researchers

**DOI:** 10.1016/j.jtauto.2022.100151

**Published:** 2022-03-06

**Authors:** Mirjam Kolev, Michael P. Horn, Nasser Semmo, Michael Nagler

**Affiliations:** a Hepatology, University Clinic for Visceral Surgery and Medicine, Inselspital Bern, University of Bern, Bern, Switzerland; b Departement of Clinical Chemistry, Inselspital Bern, University of Bern, Bern, Switzerland

**Keywords:** Laboratories / standards, Diagnostic techniques and procedures, Sensitivity and specificity, Diagnostic test approval, Predictive value of tests, Biomarkers

## Abstract

Clear guidance is needed in the development and implementation of laboratory biomarkers in medicine. So far, no standardized phased approach is established that would pilot researchers and clinicians in this process. This leads to often incompletely validated biomarkers, which can bear the consequence of wrong applications, misinterpretation and inadequate management in the clinical context.

In this conceptual article, we describe a stepwise approach to develop and comprehensively validate laboratory biomarkers. We will delineate basic steps including technical performance, pre-analytical issues, and biological variation, as well as advanced aspects of biomarker utility comprising interpretability, diagnostic and prognostic accuracy, and health-care outcomes. These aspects will be illustrated by using well-known examples from the field of immunology.

The application of this conceptual framework will guide researchers in conducting meaningful projects to develop and evaluate biomarkers for the use in clinical practice. Furthermore, clinicians will be able to adequately interpret pre-clinical and clinical diagnostic literature and rationally apply biomarkers in clinical practice. Improvement in the implementation and application of biomarkers might relevantly change the management and outcomes of our patients for the better.

## 1 Clinical vignette

An 18-year old woman was admitted during the night due to a markedly reduced general condition and shortness of breath. Her chart showed an elevated blood pressure, tachycardia and tachypnea. When I entered her room, I saw her sitting in a chair, turned towards the window. In the damp light of the morning sun, I saw pearls of sweat on the side of her face, as she was bent forward, arms on the chair handle for support, her breathing accentuated. Her legs were swollen and as I walked towards her, I noticed the visible jugular veins. The nurse that was about to leave the room told me that the urine had a foamy appearance. When I addressed the patient, she lifted her head and greeted me with a nod. This was when I noticed not only the exhaustion written on her face, but also the darker skin on the forehead of this young female with African descent. As she moved her arm for positioning, I saw the swollen wrists and MCPs on both sides. Clinical examination further revealed dampened sounds at the basis of both lungs and a friction rub on auscultation of the left lower sternal border.

Laboratory testing is the next step to confirm the suspected diagnosis. What characteristics must a laboratory test fulfill to be helpful in this situation? How does this test need to be evaluated to prove its utility?

## 2 Introduction

**Fig. 1:**
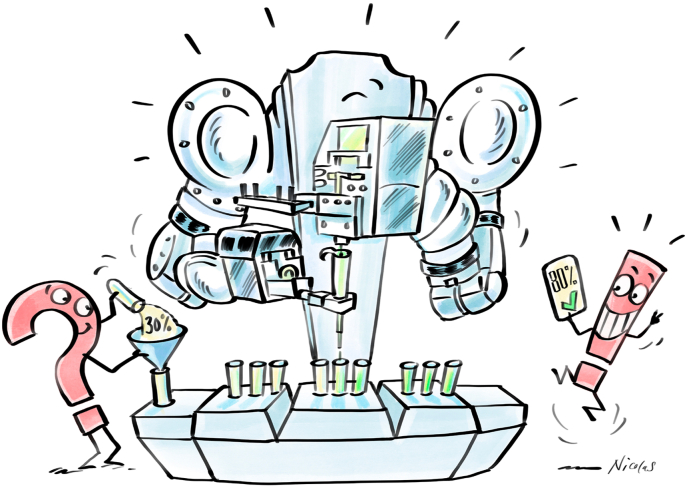
Laboratory testing answers diagnostic questions by changing the pre-test probability of a disease (e.g., the prevalence in a particular setting) into a post-test probability that supports clinical decision-making. Ideally, a certain disease can be ruled-in or ruled-out without further testing. Likewise, laboratory testing can answer prognostic questions or facilitate monitoring. The utility of laboratory tests to answer clinical questions strongly depends on the diagnostic accuracy in real-life clinical settings.

The introductory clinical case presented above illustrates the situation doctors regularly face. Following history taking and physical examination, laboratory testing is often the first central step in the diagnostic work-up. Which test to be used is based on the clinical question and probabilistic reasoning. Most often, we need an answer to a diagnostic problem, as illustrated in Fig. 1. Ideally, the disease under consideration can be confirmed or ruled-out with a single laboratory test. However, this is rarely the case in clinical practice. In contrast, clinicians must integrate various laboratory tests, and results might even be contradictory. Profound knowledge about test characteristics and clinical performance is necessary to make correct interpretation possible. This knowledge is gained in appropriately designed validation studies.

However, have we ever invested much time searching the evidence for the biomarker we are using in daily clinical practice? We are well aware of the different phases in the development and implementation of new drugs. We regularly check on potential side effects, interactions and reassure ourselves on clinical trial evidence. When it comes to laboratory biomarkers, is it even possible to find accurate and meaningful information? Which are the phases in the development and evaluation process of biomarkers? What are essential research questions to be answered before implementation?

Many years later, the introductory case is still vividly in memory with this typical presentation of a fulminant systemic lupus erythematosus. ANA and anti-dsDNA testing were performed, and they were highly positive. Not much time had to be invested in the reasoning of ANA-interpretability, pre- and-post-test probabilities or utilities, as the clinical presentation gave way to the diagnosis. More difficult is the common situation of a lower pre-test probability. People are evaluated for autoimmune diseases with mild complaints, not uncommonly only because of a positive autoantibody or symptoms, which could add up to a puzzle, leading to a diagnosis, but can as well be interpreted individually. It is often not easy to draw the line between expectative waiting, further testing or making a diagnosis and beginning a treatment. It is in these settings, where an optimization of our concepts of biomarker evaluation is crucial.

## 3 Laboratory tests are often inadequately evaluated

Currently, requirements for licensing new laboratory tests are low. In essence, manufacturers are asked to demonstrate analytical sensitivity, linearity, and precision in a limited number of samples. However, the ability of the test to answer a diagnostic or prognostic clinical question must not be demonstrated in an adequate clinical study. Besides, whether or not the test improves health-care outcomes remains open. It has been recognized that new biomarkers are implemented too early [1] and are not evaluated adequately [2].

What is the reason for the inadequate evaluation? It appears that researchers and clinicians are often not aware of the methodological tools available [3]. The research that has been done on developing methodology is heterogeneous and has not yet found its implementation in regulatory frameworks [[4], [5], [6]]. A brief overview of the existing regulations is given in Table 1. Regulations of authorities often focus on analytical precision only and the verification of the diagnostic or predictive utility of a laboratory test is not requested for licensing [3]. Besides, pre-analytical issues and biological variation is not studied adequately in many situations [7]. In particular, studies documenting improved clinical (e.g. morbidity and mortality) and healthcare outcomes (processes, costs) are largely missing. Even though improved outcomes are the ultimate aim of laboratory testing, getting these studies funded is extremely difficult. Neither industry partners nor funding agencies are interested in supporting laborious clinical studies evaluating laboratory tests.

**Table 1:** International regulations and scientific guidelines affecting autoimmune diagnostics in medical laboratories.

Type of regulation	Authority	Scope
In-vitro diagnostics regulation (IVD-R) 2017/746 [55]	European Parliament and Council	Regulation for making in-intro diagnostic medical devices available on the European market.
EN/ISO 15189/17025 [55]	European cooperation for Accreditation (EA)	Enables laboratories to demonstrate that they operate competently and generate valid results.
IVD regulations and Clinical laboratory Improvement Amendmends (CLIA) [56]	U.S. Food and drug administration	Regulate laboratory testing and require clinical laboratories to be certified by the Center for Medicare and Medicaid Services (CMS)
Eurolab cookbooks [57]	European Federation of National Associations of Measurement, Testing and Analytical Laboratories	Short documents to help laboratories comply with ISO 17025.
Guideline [58]	Clinical and Laboratory Standards Institute (CLSI)	Quality assurance of laboratory tests for autoantibodies to nuclear antigens
Guidelines for Antinuclear Antibody Testing [59]	International Federation of Clinical Chemistry	To provide guidance for antinuclear antibody testing.
Position statement [60]	American College of Rheumatology (ACR)	To specify the methodology for testing of antinuclear antibodies.
Recommendation [61]	European autoimmunity standardization initiative (EASI)/International Union of Immunologic Societies (IUIS)	To deliver a European recommendation on quality control and accreditation for laboratories involved in autoantibody testing.
Consensus statement [62]	International consensus on ANA patterns (ICAP)	Consensus regarding the morphological patterns observed in the indirect immunofluorescence assay on HEp-2 cells.

Considering that 75% of all medical decisions are somehow affected by laboratory test results [8], we can estimate the potential harm associated with inadequately validated biomarkers. Falsely positive or negative results can have profound medical (e.g. inadequate treatment decisions), emotional (e.g. anxiety), social (e.g. effects on relationships) and financial consequences for the individual patient.

## 4 Proposal for a conceptual framework

A comprehensive conceptual framework can address these problems in several ways. First, all factors affecting the utility of laboratory tests are addressed in one model and can be seen at a glance. Secondly, the framework can be used as a methodological toolbox providing study designs for all phases of the evaluation process. Thirdly, it can be used as a checklist to assess the completeness and the methodological quality of previous evaluation studies. Fourthly, the phased approach ensures that essential characteristics of new laboratory tests are determined first and that studies of a particular phase take results of the previous phase into account. In addition, the evaluation process can be stopped early in case of inadequate results. Fifthly, authorities and scientific societies can use this framework to define minimal requirements to be fulfilled before implementation of a new test. And sixthly, physicians will be informed more comprehensively about the diagnostic utility of laboratory tests in clinical practice.

Despite it being described in phases, it is important to perceive that the implementation of laboratory tests is a cyclic process that begins with the recognition of an unmet clinical need and continues through an expansive evaluation process, assessment, and adoption. It sometimes slips back to address unanticipated problems and then advances again as those are resolved, or might be stopped altogether, when results fail to support continuation of the development process.

**Fig. 2:**
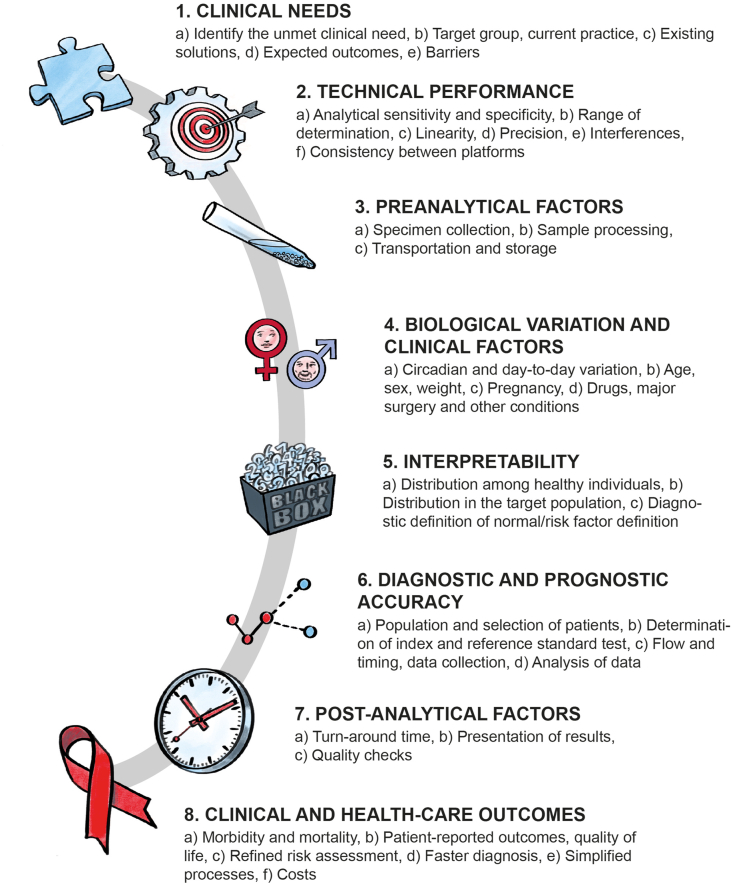
A conceptual framework for the development and implementation of laboratory tests. Adapted from Nagler M, Hämostaseologie 2020.

With the present article, we will introduce a conceptual framework, a phased methodological approach, for the development and evaluation of laboratory biomarkers, as illustrated in Fig. 2. Underlined by common examples from immunology, we will describe all aspects of this framework, detail methodological tools, and discuss possible applications in scientific inquiry and clinical practice.

## 5 The conceptual framework for the implementation of laboratory tests

### 5.1 Phase 1: defining the unmet clinical needs

In our clinical practice, there are numerous situations, in which we would wish for biomarkers, which are more accurate, more rapid, less invasive, or more tailored to specific treatments than current laboratory tests. This wish list reflects unmet clinical needs, which is the ideal starting point for the development and evaluation of a new biomarker. Assessing the unmet clinical need helps focusing on relevant health care issues, increasing the value and reducing waste in biomedical research. Four triggering questions are suggested to clarify the unmet clinical need for a proposed new biomarker [9]: (a) *What is the clinical management problem and desired outcome?* (b) *Is there an existing solution?* (c) *Would the biomarker contribute to the solution?* (d) *Is the biomarker solution feasible in practice?* This stepwise approach can be used regardless of the intended purpose (diagnosis, prognosis, and monitoring) and role within the clinical care pathway (e.g. more accurate diagnosis; replacement of an invasive test). It is evident that defining the needs of the current clinical care pathway is an essential requirement in the process of development and implementation of new biomarkers.

Platforms promoting the exchange between participating stakeholders can generate knowledge, ideas and strategies. Clinicians are daily confronted with clinical problems and help define the unmet needs. Industry partners and researchers conducting translational science draw attention to new analytical technologies. Healthcare authorities can define priorities to be solved by academic groups and industry partners. Besides, societal needs for an equally accessible healthcare system can be the driver of funding schemes and innovations.

### 5.2 Phase 2: technical performance

It seems clear that technical performance is a fundamental prerequisite for the successful implementation of any laboratory test [3]. Using an insensitive analytical technique will result in many false-negative test results. Precision and robustness are other essential characteristics that ensure reliable test results under altered conditions. Understanding the laboratory techniques and analytical performance helps to interpret the test results in clinical practice. As an example, antinuclear antibodies (ANA) can be determined by indirect immunofluorescence (IIF) on cell lines or tissue sections, or with various immunoassays. IIF done on HEp-2 cells is regarded as the highly sensitive gold standard since it can detect more than 150 autoantibody targets, which are expressed on these cells [[10], [11], [12]]. However, it stays descriptive and cannot report on the specific antigen seen (e.g., homogenous nuclear pattern without information about the targeted antigen). In contrast, enzyme-linked immunosorbent assays (ELISA) and other immunoassays can differentiate antibodies against *particular/specific* antigens searched for, such as e.g. double-stranded DNA or DFS-70 (dense fine speckled) [13]. The disadvantage of these rapid and automatable tests is that particular ANAs can easily be missed because only a small proportion of the ANA targets covered by IIF is usually requested. Therefore, the combination of the technically differently performing methodologies is mandatory to characterize the immunological process more completely. Another aspect is the observer dependence in the interpretation of IIF patterns, requiring extensive training and practice. These differences in expertise are recognized. As a strategy to make technical performance more transparent and reliable, the International Consensus on Antinuclear Antibody Pattern (ICAP) defined that some ANA patterns have to be recognized by all laboratories (mandatory level) while more complex patterns are only expected to be distinguished by experts (expert-level) [14]. Several manufacturers of substrates and reagents for ANA diagnostics have implemented fully automated computer assisted immunofluorescence reading systems. This computer assisted reading of the ANA slides might compensate lacking expertise in manual reading. However, most of the automated immunofluorescence reading systems have limited capabilities of pattern recognition [15,16].

### 5.3 Phase 3: preanalytical factors

What causes most diagnostic errors? Astonishingly, most of the mistakes leading to wrong results happen even before the blood samples arrive in the laboratory [3]. Among these mistakes, a mix-up of patients is the most relevant error, possibly leading to a delay in diagnosis or even incorrect treatment. Besides, taking the wrong tube, under filling, or too long storage times before transport are common problems. An important example in immunology is the quantification of cryoglobulins, which require strict pre-analytical conditions [17]. As another example, centrifugation schemes can have a relevant impact on laboratory tests [18]. In contrast to other laboratory medicine areas such as hemostasis, antibodies are very stable and can mainly be processed without complicated freezing procedures.

These considerations make it obvious that attention to the pre-analytical phase can have a high impact on the quality and utility of laboratory test results. Much can be achieved by implementing standardized procedures and laboratory automation as well as education of the staff involved. In addition, many unclear cases can be solved with a direct conversation between the laboratory technician and treating physician.

### 5.4 Phase 4: clinical factors and biological variation

Biomarker levels vary between males and females, newborns and adults, pregnant and non-pregnant women, over and underweight individuals and can have circadian variations [19]. These examples make it clear that biological variation of biomarkers must be considered while interpreting laboratory test results. To discuss one important issue on something seemingly obvious: men and women are biologically different. Is this adequately applied in scientific inquiry? A major gender difference has long been acknowledged in autoimmunity, as most autoimmune diseases are more prevalent in women [20]. Still, some authors argue that including both sexes in experiments is a waste of resources [21]. Females (both humans and animals) are, it is argued, too complex, too variable, and too costly to be tested on [22]. In basic research, differences between male and female cells are recognized, but most cell studies included male cells only and merely few report on sex-based results [23]. Levels of many biomarkers are different between males and females as well [24]. If we fail to include both sexes from the very beginning of our research, it is not only scientifically unreasonable, but also an ethical issue as well. It's not only about standard levels of biomarkers being incorrect in women, but we also must consider the necessity to establish new female and male-specific biomarkers.

A wide range of clinical factors affect laboratory test results. As an example in the context of autoimmunity, total IgG is an important biomarker contributing to diagnosis and monitoring of autoimmune hepatitis (AIH) [25,26]. In case of advanced liver disease, however, total IgG is often elevated as well and cannot be used anymore for the interpretation of AIH disease activity [27]. Furthermore, total IgG is elevated in associated Sjögrens’ Syndrome, which is present in 7–10% of AIH cases, again relativating the value of IgG in this clinical context [28,29]. These examples illustrate that clinical factors must be considered while interpreting laboratory test results. Furthermore, it is necessary to implement them in respective evaluation studies, anticipating confounding parameters.

### 5.5 Phase 4: post-analytical phase

Laboratory test results must be delivered timely and in a clear and easily accessible form to be useful in clinical practice. Even the best performing laboratory test will not support clinical management if the result is received too late to make the necessary decision. To give an example, acute severe autoimmune hepatitis needs rapid treatment with steroids to avoid serious complications, including liver failure. However, one must rule-out the important differential diagnosis of hepatitis E virus to enable safe treatment with steroids, since steroid treatment can be deleterious in the case of hepatitis E. Diagnosing hepatitis E depends on the antigenic PCR, which usually takes several days. Antibody assays against hepatitis E (IgG, IgM) are often false-positive in this setting [30,31]. Thus, long turnaround times might lead to serious clinical dilemmas and hazardous treatments. Another issue which might appear in the post-analytical phase is unclear reporting. To address this issue, the ICAP has published an international consensus towards harmonization [14,32]*.* Laboratories are requested to state the type of assay used (e.g. IIF or ELISA), the test result, one or more precisely defined IIF patterns and the autoantibody titer. Besides, clinical associations should be avoided as interpretations must be done in the clinical context [33].

### 5.6 Phase 5: interpretability of the test

We are taught that reference ranges are the best measure to decide whether a test result points towards a particular diagnosis or disease activity in individual patients. However, the drawbacks of the reference range approach can be illustrated by the Gaussian’ distribution of values determined in healthy volunteers, which is the statistical principle of the reference range [3]. By definition, five percent of healthy individuals will always have results outside the reference range. To make it more complex, there is also a Gaussian’ distribution of test results in diseased individuals, which often overlaps with the curve obtained in healthy individuals. As an example, diagnosing and monitoring primary biliary cholangitis (PBC) relies on an elevated alkaline phosphatase. However, histologically active PBC is found in up to 80% of patients with alkaline phosphatase within the reference range [34]. This sets patients at risk for delayed treatment and progression to cirrhosis [35].

Other approaches have been developed to address the problems of the *reference range approach*: (a) the *target cohort approach*, (b) the *diagnostic definition of normal* or *risk factor definition*, and (c) the *therapeutic definition of normal* [5]. Each of these options are all associated with certain drawbacks [3]. Using the *target cohort approach*, clinicians can differentiate patients with the disease from patients without the disease in a cohort of patients with similar symptoms using particular cut-off values. It is much more challenging to perform these studies, as they must be tested in patients with symptoms, and conducted in an adequately powered cohort. Another drawback is that some tests are used to answer several different clinical questions, which makes reporting of the test results challenging. In the *diagnostic definition of normal*, the risk of the patient of having a disease is taken into account for the definition of the cutoff. The drawback of this approach is that large and well-designed studies are necessary to obtain the estimates needed. In addition, the definition may change regularly as new studies come up. The most advanced approach would be the *therapeutic definition of normal*, where laboratory values consistent with a patient population that benefits from a certain treatment are used as a cutoff. The drawbacks of this approach are that the abnormal definition is applicable only to a certain patient population, and it is very costly to conduct the underlying studies.

However, as long as the reference range approach remains the standard approach for most situations, it is important to consider clinical characteristics and other laboratory tests to interpret the results as adequately as possible in individual patients.

### 5.7 Phase 6: diagnostic and prognostic accuracy

The most challenging question physicians are confronted with in the diagnostic process is *“What does this test result mean for my patient”*? This refers to the diagnostic utility of a laboratory test, which can only be established in adequately designed diagnostic accuracy studies. Ideally, a test increases or decreases the probability of a disease to such an extent, that we can be reasonably sure about the presence or absence of this disease and conclude the diagnostic work-up. The diagnostic performance can be quantified by the likelihood ratio (positive or negative).

The probability of the disease at a given test result (the post-test probability) can be calculated from the pre-test probability (the clinical likelihood) using the Bayes’ theorem and the positive or negative likelihood ratio [36,37].

This process is exemplified in Fig. 1. Even though the (positive or negative) likelihood ratio is the most useful diagnostic accuracy measure of a test, sensitivities, and specificities are used more frequently. The sensitivity of a test gives the proportion of diseased patients, which are identified by the respective test [38]. The lower the sensitivity, the more patients *with* the disease are missed by the test. Accordingly, the specificity gives the proportion of non-diseased individuals, which are identified by the test. The lower the specificity, the more patients *without* the disease are falsely classified as having the disease. Of note, sensitivities, and specificities are not fixed test properties but rather characteristics of the test under certain clinical circumstances (e.g., changing prevalences) [39]. Positive and negative predictive values are measures, which are very intuitive to understand but highly dependent on the prevalence. All these measures are used to describe the diagnostic utility of laboratory tests. It is worth mentioning that the results for autoantibodies do not show a Gaussian distribution.

Obviously, the utility of laboratory tests critically depends on the diagnostic accuracy to rule-in or rule-out a certain disease. And, appropriately designed diagnostic accuracy studies are required to determine valid performance measures. The diagnostic accuracy literature, however, suffers from poor study design, small study samples without power calculations, and suboptimal reporting [40,41]. In particular, the selection of the study population is a critical issue because this population defines the target population to which the test can be applied. Often, highly selected patients are used, which results in unrealistic and biased diagnostic accuracy measures [42]. Other well-known examples of poor design characteristics which lead to biased results are: (a) reference standard tests are inadequately used, (b) different conduction of the index test in contrast to clinical practice, (c) poor flow and timing of the study procedures, and (d) low sample sizes.

As an example, a young woman who has a discrete malar rash, joint complaints, and positive ANA (1:320) is seen with the suspicion of SLE. However, the rash could well be rosacea and the joint pain has not a clear inflammatory pattern. In this situation, the consecutive determination of specific autoantibodies can essentially change the probability of SLE. Her anti-DFS70 was positive and further antigen-specific ANA differentiation, including anti-dsDNA and anti-Sm antibodies, was negative. This constellation is a strong argument against SLE [43,44]. Thus, determination of specific ANA significantly decreased the post-test probability and ruled-out SLE.

### 5.8 Phase 7: utility of laboratory tests

Laboratory testing aims to support the primary goal of any medical consultation, which is improving symptoms and preventing complications. If we question the utility of a laboratory test, we have to ask whether the determination of a particular test improves clinical and healthcare outcomes. Clinical outcomes refer to morbidity, which is defined by the disease entity, and mortality. Patient-reported outcomes including intensity of pain, fatigue, and functioning can be assessed using disease-specific or generic quality-of-life questionnaires. Process outcomes describe the effects a laboratory test has on care pathways, for example, a faster or less invasive diagnostic workup or more accurate prediction of disease evolution. Another important healthcare outcome is costs. Laboratory testing can save costs or, much more frequently, increase costs on an individual and healthcare level [45]. The most rigorous study design to analyze these aspects would be a randomized controlled trial (RCT) analyzing diagnostic pathways with and without the test under consideration [46]. However, RCTs analyzing these aspects are hardly ever done. More frequently, cohort studies are employed, but the methodology is often limited. Hence, these questions generally remain unanswered.

As an example, ANA testing used to be performed mainly by rheumatologists to verify systemic autoimmune diseases. Over the years, however, ANA-testing has become popular also in low-prevalence primary care settings used in patients with wide-spread pain [47]. However, several studies analyzing the results of this management suggest significant downstream effects and negative outcomes. About 90% of patients tested for ANA in the primary care setting, which were subsequently transferred, were not diagnosed with an autoimmune disease [47]. Of note, a significant proportion of these patients already received treatment with systemic steroids [48].

## 6 Application of the methodological framework

How does this methodological framework help in the implementation and critical appraisal of laboratory tests? These structured criteria can guide physicians to assess the literature studying a particular laboratory test. Is the evaluation process complete, and is the methodological quality adequate? How significant is this test result for my patient? What does it mean in terms of diagnosis or prediction of future events?

For researchers aiming to implement new biomarkers into clinical practice, this conceptual framework can be used as a methodological toolbox. Which research questions must be answered? Which study design should be applied, and what research methods must be used? In what order should the studies be conducted? In a phased approach, essential test characteristics are determined first and studies of a particular phase take the results of a previous phase into account. Furthermore, the evaluation can be stopped early in case of inadequate study results and costs associated with more expensive later stages can be saved.

With the help of this methodological framework, knowledge obtained in evaluation studies can be summarized to assess completeness, methodological quality, and adequacy of test performance. This information can be used by scientific societies releasing guidelines on the diagnostic work-up. Besides, it can be used by laboratory managers deciding upon the implementation of new tests and health care authorities considering reimbursement.

## 7 Challenges in rare diseases

Physicians working in specialized outpatient units are confronted with rare and very rare diseases. Assessing the diagnostic value of laboratory tests to be used in these patients is extremely difficult. To avoid the high risk of bias associated with diagnostic case-control studies, a large number of patients with a *suspected* disease must be studied in order to include one patient *with* the disease. Often, the number of patients available even in large centers is insufficient for a well-powered analysis. Nevertheless, there is no getting around making good studies; it is well acknowledged that inadequate study designs result in glaringly biased results and often mismanagement of patients [42,49].

What can be done to address this problem? Obviously, collaboration is a key success factor to answer research questions in rare diseases [50]. Data obtained in various institutions can be shared using patient registries and databases collecting routine clinical data. Initiatives promoted by international societies can support these projects. Besides, meta-analytical methods can pool data on the level of already published primary studies or individual patient-data. The inclusion of patients can be supported with the help of electronic tools automatically collecting standardized clinical data from electronic records. These databases can even be complemented by routine bio banking of blood and other materials.

## 8 Future perspectives

Impressive new technologies appeared which might completely change laboratory medicine. Extremely sensitive techniques including immunological assays can identify biomarkers on the level of single molecules [51]. Current next-generation sequencing platforms analyze complete genomes in a breathtakingly short amount of time [52]. Huge databases are in development collecting clinical data, genetic information, and multi-omics biomarker results. Moreover, as an emerging field of science, artificial intelligence develops numerous techniques to combine these data and generate algorithms predicting clinical events [53]. Multivariate prediction models can provide diagnostic or prognostic information taking multiple diagnostic tests and clinical situations into account [54]. The question arises of how to use these techniques’ potentials to improve diagnostic and prognostic processes in healthcare. Methodological tools must be created, and criteria defined to develop, evaluate, and assess the quality of new diagnostic instruments. The conceptual framework described above presents a first proposal in this direction. This framework can be seen as a methodological toolbox to be used by researchers aiming to implement new diagnostic instruments. Relevant research questions can be identified, the associated study design selected, and the study protocols generated. The implementation of these studies must be encouraged by public health institutions, requested by scientific societies, funded by major funding agencies, and called by influential journals.

## 9 Conclusions

Laboratory tests are critical aspects of the diagnostic process and profoundly affect medical decisions. Thus, the diagnostic performance of laboratory tests used in clinical practice is an important aspect of the quality of care. However, assessing the diagnostic utility of laboratory tests is a difficult task for clinicians ordering laboratory tests and researchers aiming to translate new biomarkers into clinical practice. Often, laboratory tests are inadequately evaluated, potentially resulting in an inappropriate application, misinterpretation, and even harmful patient management. To address this problem, we propose a conceptual framework and a standardized approach to be used in the evaluation of new biomarkers and appraisal of existing tests. It covers the assessment of unmet clinical needs, technical performance, pre-analytical issues, biological variation, interpretability, diagnostic accuracy, and health-care outcomes. The conceptual framework can help physicians estimate the utility of laboratory tests, and it can guide researchers in implementing new biomarkers into routine clinical practice.

## Declaration of competing interests

The authors declare that they have no known competing financial interests or personal relationships that could have appeared to influence the work reported in this paper.
